# Preliminary study on non-viral transfection of F9 (factor IX) gene by nucleofection in human adipose-derived mesenchymal stem cells

**DOI:** 10.7717/peerj.1907

**Published:** 2016-04-14

**Authors:** Susana Olmedillas López, Mariano Garcia-Arranz, Damian Garcia-Olmo, Antonio Liras

**Affiliations:** 1 Health Research Institute-Jiménez Diaz Foundation (iiS-FJD), Madrid, Spain; 2 Department of Surgery, School of Medicine, Autonoma University of Madrid, Spain; 3 Department of Physiology, School of Biology, Complutense University of Madrid, Spain; 4 Victoria Eugenia Royal Hemophilia Foundation, Madrid, Spain; 5 Health Research Institute-Hospital 12 de Octubre Foundation (iiS-i+12O), Madrid, Spain

**Keywords:** Advanced therapies, Hemophilia B, Factor IX, Non-viral, Gene therapy, Nucleofection, Human mesenchymal adipose-derived stem cells

## Abstract

**Background.** Hemophilia is a rare recessive X-linked disease characterized by a deficiency of coagulation factor VIII or factor IX. Its current treatment is merely palliative. Advanced therapies are likely to become the treatment of choice for the disease as they could provide a curative treatment.

**Methods.** The present study looks into the use of a safe non-viral transfection method based on nucleofection to express and secrete human clotting factor IX (hFIX) where human adipose tissue derived mesenchymal stem cells were used as target cells *in vitro* studies and NOD. Cg-Prkdcscid Il2rgtm1Wjl/SzJ mice were used to analyze factor IX expression *in vivo* studies. Previously, acute liver injury was induced by an injected intraperitoneal dose of 500 mg/kg body weight of acetaminophen.

**Results.** Nucleofection showed a percentage of positive cells ranging between 30.7% and 41.9% and a cell viability rate of 29.8%, and cells were shown to secrete amounts of hFIX between 36.8 and 71.9 ng/mL. hFIX levels in the blood of NSG mice injected with ASCs transfected with this vector, were 2.7 ng/mL 48 h after injection. Expression and secretion of hFIX were achieved both *in vitro* cell culture media and *in vivo* in the plasma of mice treated with the transfected ASCs. Such cells are capable of eventually migrating to a previously damaged target tissue (the liver) where they secrete hFIX, releasing it to the bloodstream over a period of at least five days from administration.

**Conclusions.** The results obtained in the present study may form a preliminary basis for the establishment of a future *ex vivo* non-viral gene/cellular safe therapy protocol that may eventually contribute to advancing the treatment of hemophilia.

## Introduction

Hemophilia is a recessive X-linked hereditary disorder characterized by a deficiency of coagulation factor VIII (FVIII) in hemophilia A or factor IX (FIX) in hemophilia B, and caused by mutations in the genes coding for such factors. Incidence of the disease is 1 per 5,000 live male births for hemophilia A and 1 per 30,000 live male births for hemophilia B. With an overall prevalence of 7.7/100,000, hemophilia is considered a rare hematologic disorder (Orpha number ORPHA448) (Liras, Segovia & Gaban, 2012). The severity of the disease depends on the level of factor activity in plasma: patients with <1% of normal activity are said to have severe hemophilia, those with 1–5% of factor activity are considered to have moderate hemophilia and those with activity levels between 5% and 40% fall into the mild hemophilia group. The clinical manifestations of the disease consist of hemorrhagic episodes, chiefly in the muscles, the central nervous system and the joints (hemophilic arthropathy), which may occur spontaneously or as a result of trauma. Diagnosis of the condition should involve identification of type of hemophilia, severity of the disease and type of mutation; as well as detection of the carriers of the disease (Kadir et al., 2013).

Treatment of hemophilia, currently merely palliative rather than curative, involves intravenous administration of exogenous coagulation factors, either on demand or prophylactically, with a view to preventing the disabling articular sequelae resulting from premature hemophilic arthropathy. Factor administration increases patients’ quality of life and reduces the long-term surgical cost associated with hemophilia (Srivastava et al., 2013).

In the longer term, advanced therapies (Liras, Segovia & Gaban, 2012; Liras, 2012), gene therapy (using adeno-associated, lentiviral vectors or non-viral vectors) (Walsh & Batt, 2013) and cell therapy (through the use of adult stem cells or induced pluripotent stem cells) (Miao, 2012), are likely to become the treatment of choice for the disease. Hemophilia is considered an optimal candidate for these strategies given its monogenic origin and because a slight increase in factor levels—over 5%—can convert a severe into a mild or moderate hemophilia phenotype with a significant reduction in the risk of hemorrhagic diathesis. In addition, coagulation factors—unlike other genes—do not require precise metabolic regulation (Jazwa et al., 2013), a large variety of target cells and tissues are capable of secreting the factor, and there are a number of small and large animal models of the disease available that may lead to the development of effective strategies before results are translated into clinical practice. Specifically, hemophilia B has a favorable additional feature that makes it particularly amenable to gene therapy, namely that the size of the gene that encodes factor IX is much smaller than that coding factor VIIl, which makes it easier to manage and to include in a gene transfer vector (High, 2011).

The goal of these strategies is either to develop a curative treatment while maintaining a therapeutic level of the deficient proteins in the long term (Liras, Segovia & Gaban, 2012), or to design a treatment that palliates the severe joint-related complications of the disease (Liras, Gaban & Rodriguez-Merchan, 2012). The main purpose of this treatment is to generate a minimum factor expression level allowing patients to maintain a coagulation cascade. Development of such treatments has not been exempt from difficulties, especially as far as gene therapy protocols are concerned. At present, in the clinical trials for hemophilia, the immune response to the viral vector capsid has been observed, with an immune-mediated, AAV-capsid–induced elevations in liver aminotransferase levels, but there has been no evidence for immune responses to the factor expressed. This immune-mediated AAV-capsid-induced hepatotoxic damage, may limit the vector dose that can be administered, but the data suggest that this process may be controlled by a short course of glucocorticoids, without loss of transgene expression (Walsh & Batt, 2013).

Given the difficulties mentioned above, strategies that do not use viral vectors constitute a safer treatment alternative, even if the effectiveness and length of expression of such strategies tends to be lower (Liras & Olmedillas, 2009; Wang et al., 2013b).

Several non-viral transfection methods have been reported, from the introduction of “naked” DNA alone to the use of a series of physical methods (microinjection, electroporation, hydrodynamic injection, magnetic microparticles) or even chemical ones (cationic lipoplexes (LP-DNA)) (Wang et al., 2013b). The use of transposons has also been suggested, as these are capable of integrating with the host cell genome in a controlled way (Li et al., 2013). Nucleofection, which combines electroporation with chemical methods (nucleoprotein filaments, NPF), is the most advanced and preferred technology for non-viral transfection. This method enables the non-viral transfer of genes to the cell nucleus with high transfection efficiency in cells that do not divide or that do proliferate but at a very slow rate, as is the case of primary cells, which are particularly difficult to transfect (Zaragosi et al., 2007).

In the present study, a safe and effective gene transfection method was established using non-viral transfection based on nucleofection to express and secrete human clotting factor IX *in vitro* and *in vivo* in a murine model. Human adipose tissue-derived mesenchymal stem cells (ASCs) were used as target cells as they are easy to obtain, present with a high differentiation and self-renewal potential and secrete many of the cytokines and growth factors involved in such processes as angiogenesis, wound healing and tissue repair (Liras, 2010). These cells, which also boast anti-inflammatory, anti-apoptotic and immunomodulating properties (Pikuła et al., 2013), are excellent candidates to be genetically modified and reimplanted following *ex vivo* gene therapies and/or cell therapies. Also, ASCs do not express the MHC class II antigens, allowing allogenic transplantation of the transfected cells.

## Materials and Methods

The study was approved by the Medical Ethics Committee. Fat donors agreed to participate by written informed consent, and the experiments with animal models were performed at the Experimental Department of Biomedical Research Institute IIB-CSIC (Madrid, Spain). The protocol approved by the Animal Committee Welfare Ethics (CEBA) was followed and the rules set out in the EU Directive on experimental animals (63/2010 EU) and the Spanish legislation (RD 53/2013) were complied with.

### Isolation, culture and characterization of ASCs

ASCs were obtained from healthy donors by means of suction-assisted lipectomies (Sterodimas et al., 2012; Zuk et al., 2001). A total volume of 100–300 mL of lipoaspirate samples was collected in 60 mL syringes and processed in a sterile environment. Following two washes with phosphate buffered saline (PBS) (Sigma-Aldrich, St. Louis, MO, USA), the lipoaspirates were centrifuged at 300× g for 5 min and subsequently subjected to enzymatic digestion with 0.075% collagenase type I (Gibco^®^, Invitrogen™ Life Technologies, San Diego, CA, USA) in PBS for 60 min at 37 °C using gentle agitation. The enzyme was inactivated by adding an equivalent volume of Dulbecco Modified Eagle’s Medium (DMEM) (Gibco^®^) supplemented with 10% heat-inactivated fetal bovine serum (FBS) (Gibco^®^) and 1% penicillin/streptomycin (10,000 U/mL, 10,000 µg/mL) (Gibco^®^) (complete medium) (García-Olmo et al., 2003). The mixture was subsequently centrifuged at 300× g for 10 min and the cellular sediment was washed to remove any remainder of the enzyme. The precipitate was then resuspended in 5 mL of fresh medium and subjected to density gradient centrifugation in 4 mL of Ficoll-Paque^®^ (Amersham Biosciences, Uppsala, Sweden) at 300× g for 35 min. After several washes, the resulting cell fraction was plated in complete medium at 37 °C in a 5% CO_2_ atmosphere. In cultures reaching 70–80% confluence, the cells were released with 0.05% trypsin/ethylenediaminetetraacetic acid (EDTA) (Gibco^®^) and replated at a concentration of 5,500 cells/cm^2^. Only cell passages from 3 to 10 were used in the experiments.

For adequate growth control, cells were plated in 24-well plates at a density of 1.5 × 10^4^ cells/well and were fixed with 4% paraformaldehyde at different times of culture (24 h, 3, 7, 11 and 15 days). Once fixed and washed with PBS, the cells were stained with 0.1% crystal violet (Merck, KGaA, Darmstadt, Germany) in distilled water. The crystal violet was removed with 10% acetic acid in distilled water and the absorbance of the resulting solution was measured at 595 nm.

### Characterization of ASCs

#### Identification of cell-surface markers by flow cytometry

After being released using 0.05% trypsin/EDTA, cells were washed and resuspended in PBS in aliquots of 1 × 10^5^ cells/mL each. The following fluorochrome-conjugated monoclonal antibodies were added at saturation: CD73 (Becton/Dickinson Biosciences; BDB, San José, CA, USA); CD29 (Millipore, Billerica, MA, USA) and phycoerythrin (PE)-conjugated HLA-DR (BDB); fluorescein isothiocyanate (FITC)-conjugated CD45 (BDB), allophycocyanin-conjugated (APC) CD90 (BDB); and phycoerythrin-cyanine 5 conjugated (PE-Cy5) CD34. Following incubation for 20 min in the dark at 4 °C and successive PBS washes, up to 10^4^ events per tube were acquired in a FACScalibur flow cytometer (BDB). The characterization of ASCs by flow cytometry was performed between passages 3 and 9. These cells were only expanded in culture but neither differentiated nor transfected before staining for flow cytometry.

#### Adipogenic differentiation

Cells were plated at 1 × 10^5^ cells/cm^2^ in 6-well plates and then induced in an adipogenic medium for 14 days, which was renewed every two days. The induction medium was: a complete medium supplemented with 0.5 mM isobutyl-methylxanthine (Sigma-Aldrich), 1 µM dexamethasone (Sigma-Aldrich), 10 µM insulin (Actrapid^®^; NovoNordisk A/S, Bagsværd, Denmark) and 200 µM indomethacin (Sigma-Aldrich). Cells were subsequently cultured for five more days in a complete medium supplemented with 10 µM insulin (maintenance medium). After fixing the cells with 4% paraformaldehyde for 30 min at room temperature, lipid accumulation was visualized through Oil Red O (ORO) staining (Sigma-Aldrich) at 0.3% in 60% isopropanol for 30 min using gentle agitation (Zuk et al., 2001).

#### Osteogenic differentiation

Cells were plated at 1 × 10^5^ cells/cm^2^ in 6-well plates and then induced in an osteogenic medium for 28 days, with 60% of the medium being renewed every 3–4 days. The induction medium was: a complete medium supplemented with 0.01 µM dexamethasone, 50 µM ascorbate-2-phosphate (Sigma-Aldrich), and 10 mM *β*-glycerophosphate (Sigma-Aldrich). Following the incubation period, cells were fixed with 4% paraformaldehyde for 30 min at room temperature. The calcium-rich deposits secreted by osteocytes into the extracellular matrix were visualized by means of Alizarin Red S (ARS) staining (Sigma-Aldrich). The fixed cells were incubated with 1% ARS in distilled water at pH 4.1 in the dark for 20 min at room temperature with gentle agitation (Zuk et al., 2001).

#### Chondrogenic differentiation

Chondrogenic differentiation was induced through micromass culture techniques (Dickhut et al., 2008). For this purpose, 25 µL of a concentrated cell suspension (2 × 10^4^ cells/µL) was mixed with an equivalent volume of the commercially available Matrigel^®^ basement membrane matrix (BDB). The resulting spheres were placed at the bottom of a 1.5 mL polypropylene tube with a perforated cap, where they were allowed to gellify for 40 min at 37 °C before addition of DMEM supplemented with 1% FBS and 1% penicillin/streptomycin (10,000 U/mL, 10,000 µg/mL). After 24 h of culture, 80% of the medium was replaced with a chondrogenic medium comprising DMEM supplemented with 1% FBS, 1% penicillin/streptomycin, 6.25 µg/µL insulin, 10 ng/mL TGF-*β*1 (Sigma-Aldrich) and 50 nM ascorbate-2-phosphate. Cells were incubated in this medium for 21 days. Control spheres were cultured with 1% FBS-supplemented DMEM and 1% penicillin/streptomycin. Once differentiation took place, the Matrigel^®^ spheres were fixed in 4% paraformaldehyde for 30 min and dehydrated with xylol-alcohol washes to be subsequently embedded in paraffin. The paraffin blocks were cut by microtome into 5 µm sections, which were placed on silane coated glass slides and kept at 37 °C prior to staining. Histological sections were deparaffinized and Alcian Blue 8GX (Sigma-Aldrich) staining at 1% in 3% acetic acid was performed to detect accumulation of acid mucopolysaccharides in the extracellular matrix of micromasses. Detection of the aggrecan secreted to the extracellular matrix was carried out by immunofluorescence. To do this, histological sections were heated to 60 °C for an hour before they were deparaffinized, and autofluorescence was inhibited by means of incubation for 10 min with 4 mg/mL sodium borohydride (Sigma-Aldrich) in TBS 1X (0.05 M Tris-base and 0.15 M NaCl in distilled water) at pH 7.4–7.6. After several washes with TBS, the sections were incubated for 30 min in a blocking solution containing 5% goat serum and 0.05% triton X-100 in TBS. The human anti-aggrecan primary rabbit antibody (sc-25674; Santa Cruz Biotechnology, Santa Cruz, CA, USA) diluted at 1:50 in 1:10 blocking solution was incubated overnight at 4 °C. After several washes with TBS, a goat anti-rabbit IgG Alexa Fluor^®^ 488 (Molecular Probes^®^; Invitrogen, Carlsbad, CA, USA) conjugated secondary antibody diluted at 1:500 was incubated for 1 h at room temperature and protected from light. Sections were mounted with Prolong^®^ Gold Antifade (Invitrogen) aqueous mounting medium containing 4′,6-diamidino-2-phenylindole, dihydrochloride (DAPI).

### Non-viral gene transfection

#### Plasmids

**Figure 1:**
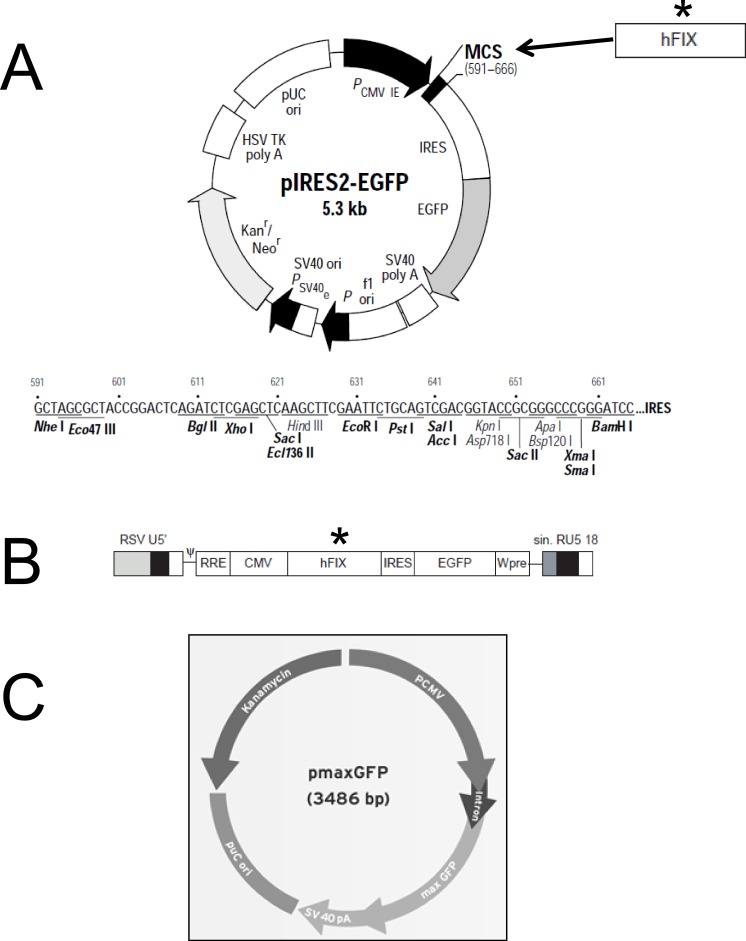
Plasmid constructions used for gene transfer studies. (A) Restriction map and multiple cloning site (MCS) of pIRES2-EGFP vector (*Clontech*). (^∗^) The DNA fragment containing intron-1 and human FIX cDNA was isolated from prrl.hFIX.IRES.EGFP and cloned into BamHI site of pIRES2-EGFP vector to obtain the pIRES2-EGFP-FIX plasmid. (B) Schematic representation of prrl.hFIX.IRES.EGFP lentiviral vector. (C) pmaxGFP vector (*Lonza*)™.

Purification of plasmidic DNA was achieved using the Qiagen EndoFree^®^ Plasmid Giga Kit (Qiagen, Hilden, Germany). The pIRES2-EGFP plasmid (Clontech) was kindly provided by Andrew F. Stewart. M.D. (School of Medicine, University of Pittsburgh, PA, USA). This vector (5.3 kb) encodes an enhanced green fluorescent protein (EGFP) and contains the constitutive cytomegalovirus (CMV) promoter which allows higher expression levels in mammalian cells (Fig. 1A). The pmaxGFP™ plasmid (Lonza) was used in the preliminary nucleofection studies to determine the method’s efficiency. pmaxGFP™ is a 3.49 kb optimized commercial construct that codes for a green fluorescent protein (maxGFP) isolated from copepod *Potellina sp.* and whose expression is controlled by the CMV promoter. Once the nucleofection protocol was standardized within the ASCs, a new plasmid was designed (pIRES2-EGFP-FIX) from vector pIRES2-EGFP, which contained the EGFP gene and an IRES sequence. The pIRES2-EGFP-FIX plasmid was obtained after cloning the human clotting factor IX gene in the pIRES2-EGFP vector (Fig. 1A). The fragment, of 1,871 pb in total, which contains intron 1 (428 pb) and factor IX cDNA (1,433 pb), was isolated from lentiviral vector prrl.hFIX.IRES.EGFP (Fig. 1B) and cloned at the BamHI site of the multiple cloning site of the pIRES2-EGFP vector.

#### Nucleofection

Of the different non-viral systems tested in our laboratory (Olmedillas et al., 2009) to determine an optimally appropriate and effective gene transfection method, nucleofection was selected as the method of choice for non-viral transfection for the target cells used in this study.

Cells subject to nucleofection must be at 85% confluence and they should have been subcultured for the last time 6 or 7 days before and with no more than 9 passages (Zaragosi et al., 2007). For each transfection, a total of 6 × 10^5^ cells were centrifuged at 200× g for 10 min at room temperature and resuspended in 100 µL of Human Mesenchymal Stem Cell Nucleofector Solution (Lonza). Subsequently, 2 micrograms of pmaxGFP™ plasmid were added to the cell suspension. As regards the transfections carried out with the pIRES2-EGFP-FIX plasmid, the amount used was 4 micrograms. The mixture was transferred to the nucleofection cuvette and subjected to program U-23 in the Nucleofector™ 2b Device (Lonza). Immediately afterwards, 500 µL of DMEM (preincubated at 37 °C under 5% CO_2_ and supplemented with 20% FBS and 1% antibiotics), was added. Lastly, cells were plated into 6-well plates containing 2.5 mL of the above mentioned medium, where they were incubated at 37 °C in a 5% CO_2_ atmosphere for 24 h before maxGFP or EGFP expression was analyzed.

**Figure 2:**
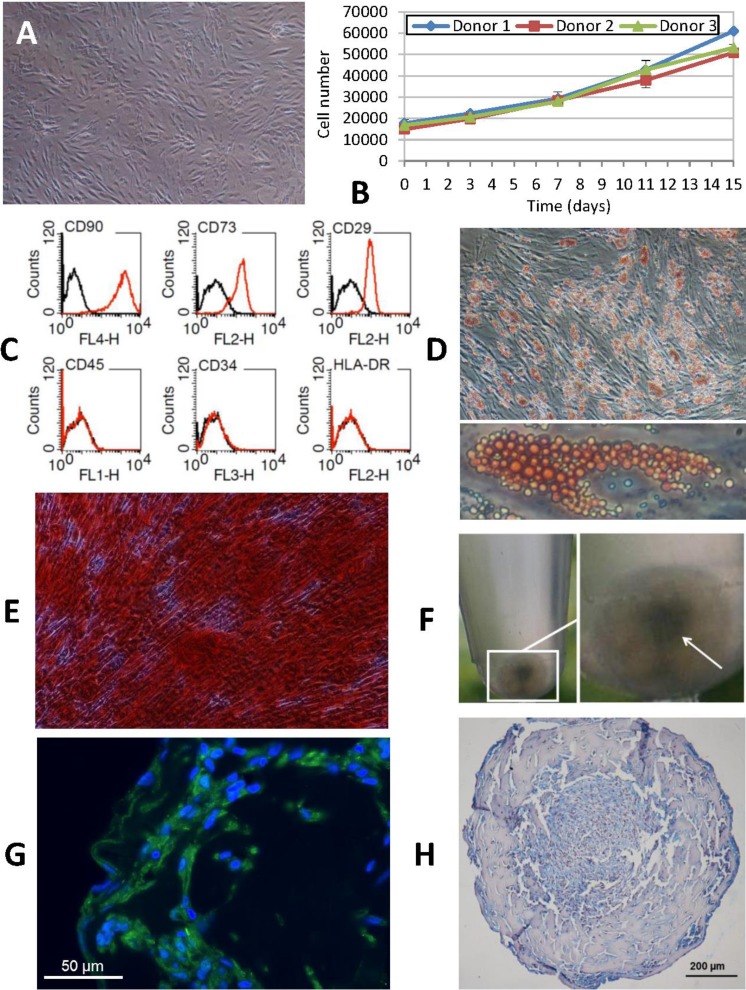
Phenotypic characterization of human ASCs. (A) Cells grown at confluence observed by contrast phase microscopy (original magnification 10×). (B) Growth curves of cell cultures obtained from three different donors showed a similar profile. The initial number of cells plated doubled after approximately 7 days in culture. Cellular proliferation capacity was preserved until the 15th day (without any passages being performed since cell plating). (C) Characterization of ASCs by flow cytometry. An analysis of the expression profile of cell surface-specific antigens showed homogeneity across all analyzed cultures: cells isolated from lipoaspirates expressed typical stromal markers such as CD90, CD73 and CD29 but not major histocompatibility complex class II antigen HLA-DR. Lack of expression of hematopoietic lineage markers CD34 and CD45 was also confirmed. (D) ORO staining shows lipid-filled droplets accumulation by ASCs upon treatment with adipogenic medium (original magnification 20×, zoomed image below). (E) Osteogenic differentiation results in a dense ARS-stained mineralized layer (original magnification 20×). (F) High density cell pellets obtained by the micromass culture technique using Matrigel™ for chondrogenic differentiation. (G) Immunofluorescence staining of aggrecan (green) in the extracellular matrix surrounding ASCs after chondrogenic induction. Nuclei are counterstained with DAPI (blue) (original magnification 40×). (H) Alcian Blue staining of micromass sections shows the accumulation of acid mucopolysaccharides in the extracellular matrix (original magnification 20×).

### Analysis of *in vitro* transgene expression and cell viability assay

Expression of transgenes by the transfected cells was analyzed at 24 h by means of fluorescence microscopy; transfection efficiency was quantified by flow cytometry. To do this, cells were trypsinized, centrifuged and washed with PBS. Finally, cells were resuspended in 400 µL of PBS and at least 2 × 10^4^ events per sample were acquired on the cytometer in order to determine cell fluorescence values. Cell viability was analyzed by a modified alamarBlue^®^ (AbD Serotec, MorphoSys UK, Oxford, United Kingdom) assay (Ahmed, Gogal & Walsh, 1994) 24 h following transfection.

### Detection of human factor IX mRNA in transfected cells

Total RNA of control cells and of nucleofected cells was extracted with TRIzol^®^ Reagent (Invitrogen™ Life Technologies) and was reverse-transcribed with the High Capacity cDNA Reverse Transcription kit (Applied Biosystems™, Invitrogen™ Life Technologies). The resulting complementary DNA (cDNA) was amplified by means of a PCR reaction using specific primers from a region of 790 bp of the gene encoding human factor IX (5′-GATGGAGATCAGTGTGAGTCCAATCCATGT-3′ y 5′-AGCCACTTACATAGCCAGATCCAAATTTGA-3′). The experimental conditions for the PCR reaction were 25 cycles of 1 min each at 95 °C, 45 s at 60 °C and 1 min at 70 °C. Parallel reactions were carried out using primers of the human *β*-actin gene as a way of ensuring homogeneous well loading (5′-GGCATCGTGATGGACTCCG-3′ y 5′-GCTGGAAGGTGGACAGCGA-3′). In these, the initial 613 bp DNA fragment was obtained by amplification at 30 cycles of 30 s at 95 °C, 30 s at 66.5 °C and 30 s at 72 °C. PCR products were analyzed by electrophoresis in 2% agarose gel and “REALSAFE nucleic acid staining” (REAL^®^ , Durviz S.L., Valencia, Spain) in a 0.5 X TBE buffer (45 mM Tris-borate and 1 mM EDTA, pH 8).

### Determination of human factor IX levels in the transfected cell culture medium and in mouse plasma

Human factor IX secreted into the culture medium by transfected ASCs, and into the plasma of mice treated with the transfected ASCs, was quantified using the ELISA technique. Samples were analyzed in duplicate using the VisuLize™ Factor IX Antigen ELISA kit” (Affinity Biologicals, Ancaster, ON, Canada) and factor IX concentrations were calculated by means of a standard curve prepared with commercially available purified human factor IX (Enzyme Research Laboratories Ltd., Swansea, United Kingdom).

### Animals

Prior to administration of transfected cells for the *in vivo* assays, and in order to stimulate “nesting” of such cells in the liver, acute liver damage was induced in the animals by administration of APAP. A preliminary study was carried out in 12 animals to search for a suitable dose of APAP to cause a mild or moderate transient and reversible damage that allowed a spontaneous recovery within a brief period of time. Mice were distributed in 4 groups (*n* = 3 per group): (1) 500 mg/kg, (2) fast + vehicle, (3) fast + 400 mg/kg and (4) fast + 500 mg/kg. Once the hepatic damage protocol was defined, the transfected human cells were injected into the mice through the tail vein. Two experimental groups were established (*n* = 6 per group), containing animals being sacrificed at 48 hours and 5 days, respectively. A control group of 3 animals subjected to liver damage without ASC treatment was included.

The NSG mice used for the *in vivo* studies are an animal model that is highly appropriate for preliminary studies where xenogenic transplants are performed. This animal model accumulates multiple defects in both their innate and adaptive immune systems. Furthermore, one of the key advantages of these types of mice, as compared with other kinds of immunodeficient mice, is that they do not develop thymic lymphoma and have a longer life expectancy. Their characteristics make NSG mice an excellent model for xenogenic transplantation as they accept heterologous cells much more easily than other immunodeficient models and, more importantly, they are an extremely efficient recipient of human cells, allow higher rates of grafting success and *in vivo* cell proliferation, and exhibit a greater capacity to differentiate into several lineages (McDermott et al., 2010).

### Induction and evaluation of liver damage in the mice

Acute liver injury was induced by an injected intraperitoneal dose of 500 mg/kg body weight of acetaminophen (APAP) (Sigma-Aldrich, St. Louis, MO, USA), established on the basis of a dose–response curve. APAP was dissolved in a mixture of propylene glycol (1,2-propanediol, Panreac Química S.L.U., Barcelona, Spain) with 50% (v/v) distilled water (50 mg/mL, 10 ml/kg). Mice were fasted for 18 h before administration of either APAP or vehicle (control group). Fasting was extended to 3 h post injection (Harrill et al., 2009). One group of mice was given the same dose of the drug without previous fasting. Water was provided *ad libitum* throughout the study.

In order to evaluate liver damage, blood was extracted through cardiac puncture under inhalation anesthesia with 2% sevoflurane (Sevorane^®^; Abbott Laboratories S.A., Madrid, Spain). Serum activity of alanine aminotransferase (ALT) and aspartate aminotransferase (AST) hepatic markers was measured with a photometric enzymatic test system (Olympus AU5400^®^; Beckman Coulter^®^, Madrid, Spain). For the histological examinations, fresh livers were fixed in 10% buffered formalin, dehydrated and embedded in paraffin. Detailed procedures have been described elsewhere (Ji & Kaplowitz, 2003). After paraffin embedding, 5-µm sections were prepared and stained with hematoxylin-eosin. Liver sections were subjected to Periodic Acid-Schiff Staining (PAS) as described in the literature (Burra et al., 2012) in order to confirm the presence of acute damage caused by APAP overdose.

### Administration of ASCs

Administration of transfected cells was carried out in 10–14 week-old NSG male mice subjected to liver injury 24 h prior to perfusion of the transfected cells. After gene transfection, cells were released with 0.05% trypsin/EDTA and washed with PBS. Subsequently, 1.5 × 10^6^ cells were injected into the caudal vein suspended in 150 µL of PBS. At 48 h and 5 days, the blood was extracted through cardiac puncture under inhalation anesthesia with sevoflurane and collected in tubes containing 3.8% sodium citrate. At the same time, the liver, the lungs, the spleen and the kidneys were excised and washed with 0.9% NaCl. The liver was divided into its lobes, some of which were quickly frozen in liquid nitrogen. One lung, one kidney and half of the spleen of each animal were also frozen and subsequently stored at −80 °C. The contralateral lung and kidney, the other half of the spleen and the remaining liver lobes were fixed in 10% formaldehyde for posterior embedding in paraffin.

### Detection of human factor IX mRNA in murine tissue

The liver, lung, spleen and kidney fragments that had been frozen at −80 °C were homogenized in a mortar with liquid nitrogen in order to extract total RNA and perform the RT-PCR reaction with specific primers from a region of 593 bp of the human factor IX encoding gene (5′-TTCTAAGCTCACCCGTGCTG-3′ and 5′-GAAGACA TGTGGCTCGGTCA-3′) using cDNA from ASCs transfected with pIRES2-EGFP-FIX as a positive control. Parallel reactions were performed using specific primers of a 442 bp fragment of the *β*-actin gene of the mouse (5′-GCTTCTTTGCAGCTCCTTCG-3′ and 5′-GCTGGGGTGTTGAAGGTCTC-3′). Primers were designed using the PrimerBlast software (www.ncbi.nlm.nih.gov/tools/primer-blast/) and synthesized by Metabion (Metabion, Martinsried, Germany). The experimental conditions for the PCR reactions were 35 cycles of 30 s at 94 °C, 30 s at 60 °C and 30 s at 72 °C. PCR products were analyzed by agarose gel electrophoresis.

### Detection of GFP-positive cells in murine tissue

Histological liver, lung, spleen and kidney sections were heated to 60 °C for an hour before subjecting them to the deparaffinization and rehydration procedure required for immunofluorescence analysis. The primary antibody used was mouse IgG2a anti-GFP (B-2) (sc-9996; Santa Cruz Biotechnology, Santa Cruz, CA, USA) diluted at 1:50 in a 1:10 blocking solution (overnight at 4 °C) and the secondary antibody was Alexa Fluor^®^ 555-conjugated mouse anti-IgG (Molecular Probes^®^; Invitrogen, Carlsbad, CA, USA) diluted at 1:500 (incubated 1 h at room temperature). After several TBS washes, a Prolong^®^ Gold Antifade (Invitrogen) aqueous medium was used to observe the final result through a fluorescence inverted microscope.

## Results

### Characterization of human adipose tissue-derived mesenchymal stem cells

At twenty-four hours from isolation, ASCs obtained from human lipoaspirate samples (Fig. 2) exhibited a rounded shape and a smaller size than those observed in subsequent subcultures. In the course of the first few days of culture, cells adopted the typical fibroblastic shape of mesenchymal cells with an enlarged cytoplasm and a more elongated appearance. An *in vitro* cell proliferation analysis was carried out where growth curves showed a similar profile across all the cultures, obtained from different donors (Fig. 2B). At time 0, cell density was 1.5 × 10^4^, which was taken as the starting point for the growth curve. Later, during the phase following adaptation of the cells to the culture conditions, a slow but continuous proliferation was observed in the studied populations. The initial number of cells plated doubled after approximately 7 days in culture, which coincided with the time needed to reach subconfluence. Moreover, cellular proliferation capacity was preserved until the 15th day (without any passages being performed since cell plating) in spite of the fact that the recommended degree of confluence had been exceeded.

Flow cytometry analysis of the cell populations obtained from lipoaspirates from three different donors was carried out in subcultured cells (passages 3 to 9). Constituting a homogeneous population, cells distributed evenly according to their size and complexity, with the same pattern being observed across all samples analyzed. An analysis of the expression profile of cell surface-specific antigens showed homogeneity across all analyzed cultures. Cells isolated from lipoaspirates expressed typical stromal markers such as CD90, CD73 and CD29 but not antigen HLA-DR of the class II major histocompatibility complex. Lack of expression of hematopoietic lineage markers CD34 and CD45 was also confirmed in all cases studied (Fig. 2C).

Characterization of ASCs was completed by looking into their adipogenic, osteogenic and chondrogenic differentiation capacity. Adipogenic differentiation was established by observing the aggregation of lipids in small rounded refringent vesicles distributed all over the cell cytoplasm in cultures containing an adipogenic medium; the number of vesicles increased as differentiation progressed. Finally, differentiation was confirmed by Oil Red O staining: triglyceride inclusions that had not been observed in the control cells appeared highlighted in red, with these round red structures taking up most of the cytoplasm of differentiated cells (Fig. 2D).

Osteogenic differentiation was evaluated by assessing the presence of calcified deposits using Alizarin Red staining. At 28 days, when differentiation was completed, cells reached a degree of overconfluence which, in the case of cultures treated with a differentiation medium, resulted in progression towards a highly marked fibroblastic shape and the formation of cell bundles where all the cells pointed in the same direction (Fig. 2E).

Chondrogenic differentiation was tested using micromass cultures with Matrigel^®^ in a chondrogenic medium. At 21 days of culture, results were positive for the secretion of aggrecan and other cartilage mucopolysaccharides, especially in the highest-density regions of the spheres (Figs. 2F–2H).

### Non-viral gene transfection

**Figure 3:**
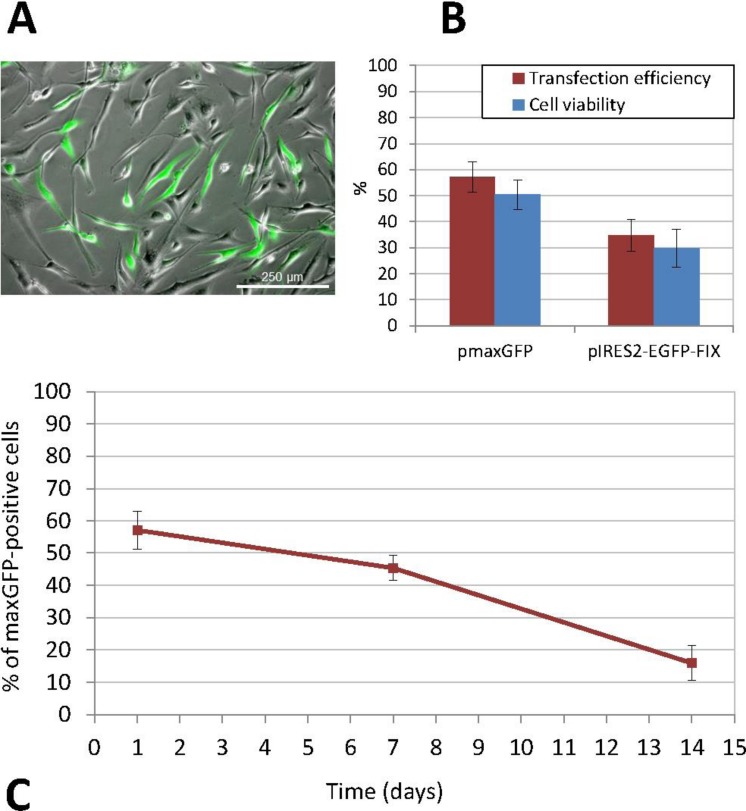
Transfection of human ASCs by nucleofection. (A) GFP expression in ASCs transfected with pIRES2-EGFP-FIX plasmid, detected by fluorescence microscopy (original magnification 10×). (B) Percentage of viable and GFP-positive cells 24 h after nucleofection with pmaxGFP and pIRES2-EGFP-FIX. Mean ± standard deviation (SD) values are represented. Both efficiency and viability were higher for the commercially available plasmid (pmaxGFP™) supplied by the nucleofection kit manufacturer (C) Time-course analysis of GFP expression in nucleofected ASCs using pmaxGFP™ plasmid. The percentage of fluorescent cells stood at 45.41% ± 3.95 at 7 days from transfection and 15.92% ± 5.45 at 14 days.

The non-viral transfection method used was nucleofection, which provided the best results both in terms of efficiency and cell viability (Fig. 3). ASCs were subcultured several times in order to increase the cell number before transfection. When we obtained a sufficient number of cells, undifferentiated cells between passages 3 and 9 were transfected. Thus, the percentage of positive cells following transfection with the pmaxGFP™ plasmid, as determined by flow cytometry, was 57.10% ± 5.92 and cell viability ranged between 44% and 55% (mean ± standard deviation, SD: 50.40% ± 5.69). Furthermore, the percentage of recovered cells, calculated as the ratio of the number of cells that had adhered to the plate 24 h after transfection to the initial number of cells exposed to nucleofection, was 40.28% ± 4.71 (mean value obtained on the basis of the results of three experiments performed in triplicate). The stability and persistence of transgene expression over the period elapsed since transfection were determined by calculating the percentage of fluorescent cells, which stood at 45.41% ± 3.95 at 7 days and 15.92% ± 5.45 at 14 days from transfection (Fig. 3C).

Similarly, nucleofection with the pIRES2-EGFP-FIX plasmid vector produced easily identifiable cells, which showed different intensities of green on epifluorescence microscopy. In this case, the percentage of positive EGFP cells ranged between 30.72% and 41.86%, (mean ± SD: 34.84% ± 6.11). The mean cell viability rate was 29.84% ± 7.16 at 24 h from transfection. Figure 3B shows a comparison between the results of transfections with pmaxGFP™ and those with pIRES2-EGFP-FIX at 24 h from nucleofection, which indicates that both efficiency and viability were higher for the commercially available plasmid (pmaxGFP™ ) supplied by the nucleofection kit manufacturer.

Cells transfected with the pIRES2-EGFP-FIX plasmid were trypsinized at 24 h from nucleofection and samples were taken before these cells were injected into the animals in order to determine the number of EGFP positive cells present by flow cytometry. Thus, it was determined that of the total number of cells perfused into each animal (1.5 × 10^6^ cells), only 32.29% ± 5.86 were EGFP positive (mean value obtained based on the results from five experiments) and carried the factor IX gene.

### *In vitro* expression of human FIX by nucleofected ASCs

**Figure 4:**
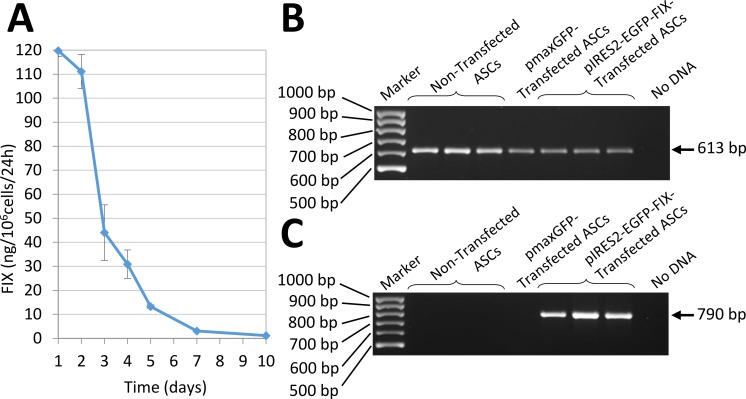
Human FIX expression by nucleofected ASCs. (A) Evolution of human FIX levels in ASC culture media after nucleofection as determined by ELISA over a 10-day period. Results shown correspond to transfected cells from the donor that secreted the largest amount of FIX. (B) RT-PCR analysis showing human *β*-actin expression, used as a control. Bands are observed in both transfected and non-transfected cells, as expected. (C) Expression of human factor IX is detected in cells nucleofected using pIRES2-EGFP-FIX plasmid but remains undetectable both in non-transfected cells and in pmaxGFP™ transfected cells.

*In vitro* expression of human FIX by ASCs transfected by nucleofection with the pIRES2-EGFP-FIX plasmid was assessed by RT-PCR (Fig. 4). Also, factor IX being a secretion protein, ELISA was used to study human factor IX levels secreted to the culture medium in order to determine factor IX expression in cells transfected with the pIRES2-EGFP-FIX plasmid. Thus, cells obtained from different donors were shown to secrete amounts of factor IX between 61.43 and 92.25 ng/10^6^ cells/24 h (mean ± SD: 74.08 ± 16.15 ng/10^6^ cells/24 h). The mean value was obtained on the basis of at least three independent experiments conducted in triplicate with cells isolated from three different donors. FIX concentration was undetectable in the culture medium of non-transfected cells (0.03 ± 0.02 ng/10^6^ cells/24 h).

Interestingly, the transfected cells obtained from one of the donors secreted a larger amount of FIX:119.77 ± 2.47 ng/10^6^ cells/24 h (mean value obtained on the basis of the results of two experiments performed in triplicate). In this case the evolution of factor IX levels in the culture medium was followed up for 10 days (Fig. 4A).

### *In vivo* studies

#### APAP-induced acute liver damage

**Figure 5:**
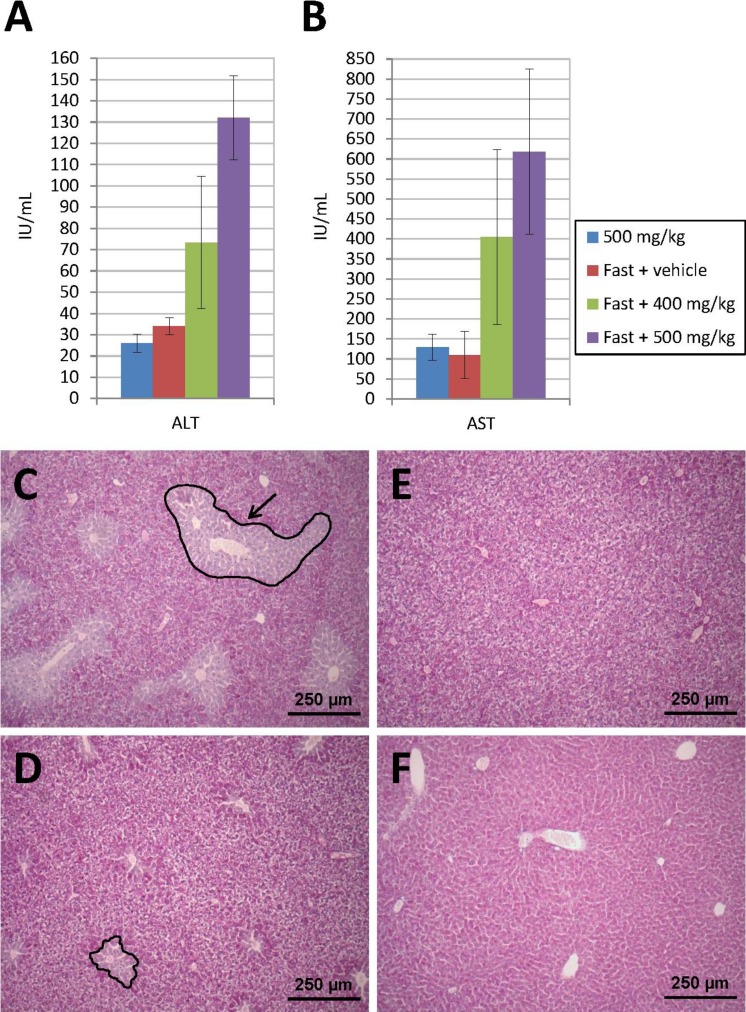
Acute liver injury induced by acetaminophen (APAP) overdose. Serum ALT (A) and AST (B) levels determined 24 h after intraperitoneal injection of APAP. Transaminase elevation is dependent on previous fasting. Animals that enjoyed free access to food did not experience an increase in ALT and AST. Those subjected to an 18-h fasting period showed a dose-dependent transaminase elevation. (C) PAS-stained liver sections show hepatic injury only in animals treated with 500 mg/kg of APAP after a fasting period. A damaged area is indicated by a black line with an arrow. (D) Lesion diameter is much smaller when APAP dose is 400 mg/kg. (E) Control fasted animal only treated with vehicle. (F) Liver section from a fed mouse treated with 500 mg/kg of APAP, where liver injury is not present (original magnification 10×).

Prior to administration of transfected cells for the *in vivo* studies, and in order to stimulate “nesting” of such cells in the liver, acute liver damage was induced in the animals by administration of APAP (see ‘Methods’ section). Figures 5A and 5B show the levels of hepatic marker enzymes ALT and AST, as determined 24 h after administration of APAP, during the preliminary tests carried out in animals subjected to acute liver damage. Thus, it was observed that, at an APAP dose of 500 mg/kg, transaminase elevation occurred as a function of whether a previous fasting period was enforced or not. Animals that enjoyed free access to food did not experience an increase in the amount of enzymes released into their bloodstream unlike those subject to an 18-hour fasting period which showed a dose-dependent transaminase elevation.

A histological examination of the liver using PAS staining was conducted. Twenty-four hours after the APAP injection, the fasted animals presented with liver damage, characterized by the loss of glycogen around the centrilobular area (Figs. 5C–5F). This area was lighter in color than the healthy tissue, where staining was more homogeneous and intense. The diameter of the pathological area was much smaller in animals treated with a lower APAP dose (400 mg/kg). In mice that received 500 mg/kg of APAP without prior fasting, the tissue appeared to be very similar to that of control animals. In the sections stained with hematoxylin-eosin, the lesion was observed in region 1, around the centrilobular area, but no involvement was identified in regions 2 and 3, where the liver parenchyma appeared intact. The pathological area was in all cases smaller than the healthy area and, in spite of the loss of glycogen, the hepatic tissue did not show any necrosis, which usually manifests itself through a mild eosinophilic stain.

#### Determination of human FIX levels in the blood of mice treated with transfected ASCs

**Figure 6:**
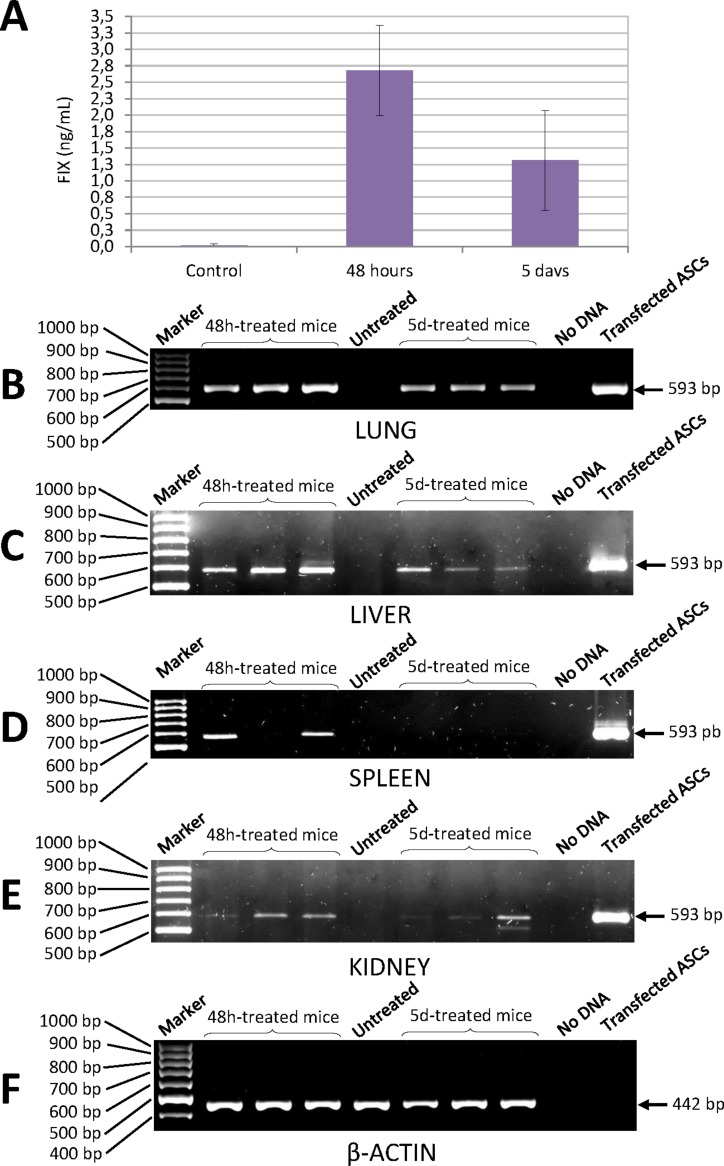
Human FIX expression in mice treated with nucleofected ASCs. (A) Levels of human FIX determined by ELISA in plasma of either control or cell-treated mice at different time points. Untreated control group was made up of 3 animals (0.02 ± 0.01 ng/mL). Concentration of human FIX was 2.68 ± 0.69 ng/mL 48 h after injection (*n* = 6) and 1.31 ± 0.76 ng/mL at 5 days (*n* = 6). (B) Human FIX expression was detected by RT-PCR in lung, (C) liver, (D) spleen and (E) kidney of NSG mice. (F) Representative image of mouse *β*-actin expression in lungs. *β*-Actin-control was performed in all tissues where Factor IX was also analysed. Lane 1: 100 bp DNA marker. Lanes 2–4: DNA from tissues of treated mice 48 h after cell injection. Lane 5: DNA from tissue of a control mouse without cell treatment. Lanes 6–8: DNA from tissues of cell-treated mice 5 days after injection. Lane 9: negative control (no DNA). Lane 10: DNA from ASCs transfected with pIRES2-EGFP-FIX plasmid.

Quantification of human factor IX levels in the blood of NSG mice injected with ASCs transfected with the pIRES2-EGFP-FIX plasmid was carried out using a highly specific ELISA kit. Concentration of human FIX was 2.68 ± 0.69 ng/mL 48 h after injection and 1.31 ± 0.76 ng/mL at 5 days (Fig. 6A). No cross-reaction was observed with murine FIX. The level of human FIX in untreated control was 0.02 ± 0.01 ng/mL (mean value based on the results from three mice).

### Determination of human factor IX mRNA in murine tissue

Expression of human FIX mRNA in the different tissues of the NSG mice treated with transfected ASCs was determined by RT-PCR. Analysis of PCRs with agarose gel electrophoresis showed an expected amplified fragment corresponding to the human factor IX gene in the different tissues (lung, liver, kidney and spleen) of the NSG mice treated, at 48 h and at 5 days from injection of the cells (Figs. 6B–6F). The said fragment corresponded to the one obtained from human cells transfected with pIRES2-EGFP-FIX, which were used as positive controls. The primers used to amplify the human factor IX gene proved to be highly specific for all the tissues studied and no cross-reaction with murine factor IX was observed.

### Detection of EGFP positive cells in murine tissue

**Figure 7:**
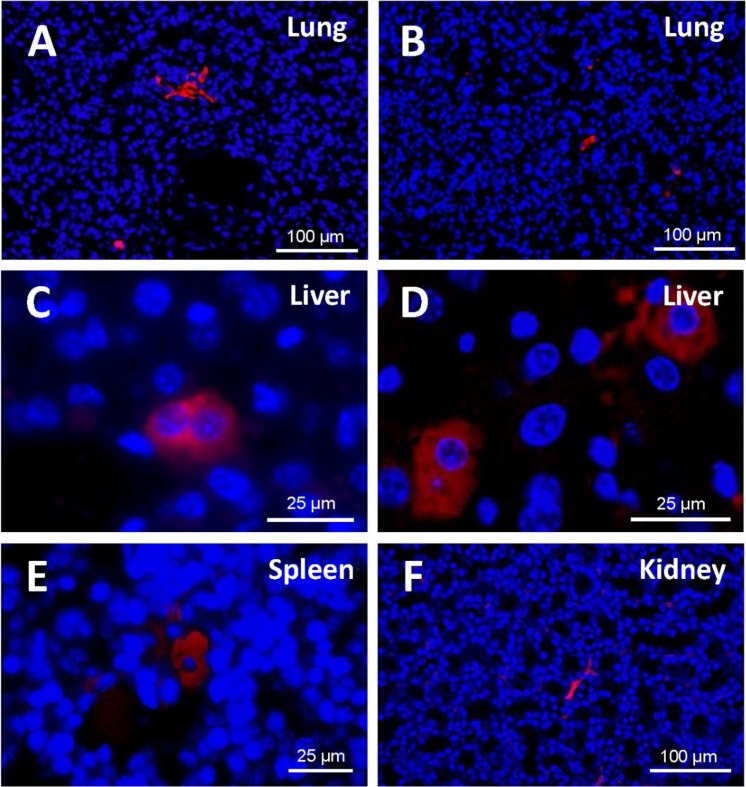
Detection of EGFP-positive cells (red) in tissue sections from cell-treated mice using immunofluorescence. Nuclei are counterstained with DAPI (blue). (A) Cell masses located in lung sections at 48 h and (B) 5 days after cell injection (original magnification 20×). At 48 h from injection, EGFP-positive cells were to be found mostly in the lung, in the form of clusters. A slight decrease in the number and size of those cell aggregates occurred at 5 days. (C) Binucleated cells expressing EGFP are observed in the liver (48 h). Cell morphology varied depending on the host tissue. (D) Liver-engrafted positive cells often show a hepatocyte-like morphology (5 days) (zoomed images from an original magnification 63×). In some mice, EGFP-positive cells are found in spleen (E) and kidney (F) (original magnification 63×and 20×, respectively). Fluorescent cells in the spleen adopted a polygonal appearance, whereas those in the kidney preserved the fibroblastic shape that characterizes ASCs.

A study of the distribution of cells in the different tissues (Fig. 7) demonstrated that, at 48 h from injection, EGFP-positive cells were to be found mostly in the lung, in the form of clusters. Being made of spongy tissue, cell aggregates took up the free spaces in the lung parenchyma. At five days from injection, a slight decrease in the number and size of the said cell aggregates occurred.

In the histological liver sections, the amount of positive cells at 48 h was much lower than in the lung; the marked cells had lost the fibroblastic shape that characterizes cultured human ASCs; and their appearance was different from that of the cell aggregates present in the lung. At 5 days, in the liver parenchyma, transfected cells increased and adopted a hepatocyte-like shape; in many cases they exhibited two nuclei, and were generally located in the vascular periphery.

EGFP-positive cells were also identified in the spleen and the kidney, although to a lesser extent. Cell morphology varied depending on the host tissue. Thus, fluorescent cells in the spleen adopted a polygonal appearance, whereas those in the kidney preserved the fibroblastic shape that characterizes ASCs.

In general, the structure of the host tissue affected the size and shape of the integrated cells. Thus, the cytoplasm of the cells found in organs such as the lung and the kidney sent out extensions and was larger than those pertaining to cells “nested” in more compact organs such as the liver or spleen.

## Discussion

The present study discusses the combination of an *ex vivo* gene therapy strategy with a cell therapy strategy, whereby adult human adipose tissue-derived mesenchymal stem cells are transfected with the human factor IX gene and then implanted in an experimental animal model. According to the latest research into cell therapy, stem cells present the advantage of high survival and self-renewal rates, which sets them apart from the fibroblastic cells used in the first non-viral gene therapy trials in hemophilia, whose results were inconclusive (Roth et al., 2001). Thus, MSCs isolated from human umbilical cord blood have already been used to over-express human FIX *in vitro* by transduction with a lentiviral DNA vector (Dodd et al., 2014).

The ASCs used in this study meet all the criteria required for potential clinical application, such as widespread availability through minimally invasive procedures, the capacity to differentiate into multiple cell lineages in an adjustable and reproducible manner and the possibility to conduct safe and efficient autologous or allogenic transplants (Pikuła et al., 2013). By the cell isolation protocol that we have here established, a homogeneous cell population has been obtained fulfilled all the requirements for mesenchymal stem cells set down by the International Society for Cellular Therapy and the International Federation for Adipose Therapeutics and Science (Dominici et al., 2006; Bourin et al., 2013).

As regards the gene transfection method selected for the study, we chose to use non-viral vectors as they appeared to be safer than viral ones in terms of their immunogenic and teratogenic response, although it must be acknowledged that their efficiency is lower and transgene expression time shorter than those of viral methods. We eventually selected nucleofection as the method of choice for non-viral transfection of the target cells used in this study, since it is a method that allows higher efficiency rates in primary cells, which are often refractory to conventional non-viral transfection systems, and particularly in bone marrow and adipose tissue-derived mesenchymal stem cells. The results obtained using this nucleofection technique were highly satisfactory, with transfection efficiency reaching almost 60% for the optimized pmaxGFP™ plasmid, a percentage that is in line with the findings of Zaragosi et al. (2007).

According to the literature (Zaragosi et al., 2007), nucleofection is an ideal transfection method for genes expressed by secretion proteins such as factor IX. Our results showed, for three different donors, that the rate of FIX secreted into the culture medium by nucleofection, ranges between 61.43 and 92.25 ng/10^6^ cells/24 h with a high degree of variability. FIX levels secreted by ASCs in this study are lower than values obtained from lentiviral transduced human umbilical cord blood-derived MSCs (4 µg/10^6^ cells/24 h) (Dodd et al., 2014) and FVIII produced by retroviral transduced bone marrow derived MSCs (292 ng/10^6^ cells/24 h) (Wang et al., 2013a). Both the FIX secretion rate and the variability observed in our study are consistent with those reported in the literature for other secretion proteins using nucleofection (Zaragosi et al., 2007). Future alternatives to increase stability of expression and reduce variability may include a combination of nucleofection with genome integration systems such as phage phiC31 integrase (Keravala et al., 2008), or the use of transposons (Matteo et al., 2012). Nucleofection has the advantage of transfecting cells that do not divide or which proliferate at a very slow rate, as is the case of mesenchymal stem cells. A functional analysis of FIX activity in cell supernatants of nucleofected cells was initially attempted using an aPPT assay. This coagulometric assay was performed in the Department of Hemostasis at La Paz University Hospital and it was found that fetal bovine serum (FBS) present in the cell culture medium of ASCs interfered with the results. In all cases measurements were above the upper limit of the working interval of the technique (>148%). Unfortunately, FBS could not be removed from the culture medium as it would be detrimental for cell viability during the first 24 h after nucleofection.

Infusion of hematopoietic progenitor cells in NSG mice is often preceded by conditioning therapy, generally based on radiation agents or on the administration of drugs like busulfan (Choi et al., 2011) intended to cause a reduction in the host’s bone marrow cells, thus providing the injected cells with a proliferative advantage. In contrast, when the goal of transplantation is the “nesting” of donor cells in the liver, conditioning therapy is often aimed more specifically at causing acute liver damage, so that infused cells can make their way to the liver in order to participate, directly or indirectly, in the tissue repair process promoted by either partial hepatectomy (Kaibori et al., 2012), or hepatotoxic drug treatment (Kato et al., 2013).

In line with previous research using the same type of biological model, in the present study mice were pre-conditioned with intraperitoneal acetaminophen (APAP) as described by Harrill et al. (2009). This protocol includes an 18-hour pre-treatment fasting phase (prior to APAP administration) followed by a further 3-hour fasting phase post-treatment.

With respect to the histological analysis, PAS staining revealed that, unlike the (also fasted) control animals, mice treated with APAP suffered a loss of glycogen in the centrilobular region, whose diameter grew as a function of increasing the dose of the drug. Loss of glycogen and microvascular damage are early histopathological lesions that occur even before transaminase levels begin to rise and generally precede the development of necrosis (Botta et al., 2006). Our goal was to cause a mild or moderate transient and reversible damage that allowed a spontaneous recovery and occurred within a brief period of time. The said damage was intended to trigger the regeneration of hepatic tissue so as to promote migration of the transfected ASCs towards the liver, where a cellular niche could be created.

Stem cells have been reported to migrate towards damaged tissues. In this study, human cells were injected into NSG mice and liver damage was planned to act as a chemoattractant of cells in our experimental model. However, induction of liver damage is not feasible in future translation to the clinical practice. Thus, alternative approaches such as intrahepatic injection of genetically-modified ASCs have been considered for future study and are at present in the development and establishment phase.

Once the hepatic damage protocol was established, the transfected human cells were injected into the mice through the tail vein. Intravenous delivery and, specifically, tail vein injection is the most common cell therapy administration method in NSG mice (Varga et al., 2010) as it is a reproducible, accessible and minimally invasive method, which requires neither anesthesia nor surgery. The dose of human cells to be injected and the intravenous perfusion rate (1.5 × 10^6^; 10 µL/5 s) were adjusted as previously reported for immunodepressed mice (Ra et al., 2011).

Human factor IX was detected in the mice’s plasma both at 48 h and at 5 days from cell administration, although levels declined with time because the nucleofection system is a non-integrative non-viral transfection method, which results in transient gene expression. At 48 h, EGFP-marked cells were located mainly in the lung, where they formed small clusters; at 5 days the presence of the cellular masses decreased slightly. This finding coincides with previous reports, which have shown that mesenchymal stem cells get rapidly retained in the lung once they are infused through a peripheral vein. After one week, the cells disappeared from the lungs and only around 1.5% of the initially injected cells maintained a stable presence in the liver.

After 48 h, although most cells in our study remained trapped in the pulmonary capillaries, a significant proportion escaped and migrated to the liver and the spleen, which is consistent with previously cited reports (Gholamrezanezhad et al., 2011). The cell barrier at the level of the lung can be attributed to the combined effect of cell size and the expression of adherence molecules in the cell membrane. Furthermore, it has been shown that the infusion of smaller cells such as mononuclear bone marrow cells, whose size is 7 µm as compared with 15–19 µm for MSCs, results in a 30-fold increase in the amount of cells crossing the pulmonary barrier with respect to MSCs (Fischer et al., 2009).

In our studies, cells initially located mainly in the lung, which could be responsible for the levels of FIX found in plasma in the early stages, later migrate to other tissues, especially to the liver, where they engraft and keep on contributing to the maintenance of existing FIX plasma levels.

Formation of a cellular niche in host tissues requires adaptation of the perfused cells, which entails an obvious change in their morphology. In the liver parenchyma, transfected cells acquire a shape similar to that of hepatocytes, and the appearance of binucleated cells suggests that some human ASCs could have merged with the hepatocytes of the host organ, an occurrence already reported for the same type of animal model (Fujino et al., 2007). However, it cannot be completely ruled out that the presence of multinucleated cells may originate from nucleofection itself, as cell fusion is a well-known effect of electroporation, a phenomenon whose frequency is proportional to the number of cells subjected to an electric pulse (Kaibori et al., 2012). Nor can the (controversial) possibility that perfused cells may undergo transdifferentiation be ignored (Follenzi et al., 2012). It would be necessary to conduct a more in-depth analysis of the EGFP-positive cells located in the liver of transplanted mice in the present study in order to determine whether cell fusion or transdifferentiation phenomena have really taken place as a result of the “nesting” of the cells in the tissue.

FIX functionality could not be analyzed in ASC-treated NSG mice as this strain does not suffer from FIX deficiency and shows normal blood coagulation. After this first set of experiments with NSG mice to establish basic protocols, future studies using the FIX knockout mouse model will be performed in order to assess the therapeutic efficacy of our approach.

In conclusion, the present study has investigated a simple and reproducible method to obtain and characterize human adipose tissue-derived mesenchymal stem cells originating from lipoaspirates. Nucleofection was the non-viral method used to transfect those cells with a plasmid so that they could be used as a gene expression vehicle when they were perfused in immunodeficient mice.

The results obtained in the present study may form a preliminary basis for the establishment of future *ex vivo* non-viral gene/cellular therapy protocols that may eventually contribute to advancing the treatment of hemophilia. Future work should center on achieving a more stable expression of the transgene in the medium and long term and evaluating the performance and biosafety of the injected cells. Efforts should also be directed at optimizing the plasmid vector through high polyadenylation sequences and endogenous or specific tissue promoters with low CpG content. Also, nucleofection may be used in combination with genome integration systems such as phage phiC31 integrase or transposon-based systems. General optimizations are likely to be based on transfectant selection, the use of alternative cell administration methods such as intrahepatic injection, and the use of biological scaffolds intended to facilitate “nesting” and long-term maintenance of the factor IX-producing cells.

## Supplemental Information

10.7717/peerj.1907/supp-1 Data S1 Raw data

10.7717/peerj.1907/supp-2 Supplemental Information 2 Primers design

## Additional Information and Declarations

### Data Availability

The following information was supplied regarding data availability: The raw data are provided as Data S1.
