# Supplementary material for: Preliminary study on non-viral transfection of F9 (factor IX) gene by nucleofection in human adipose-derived mesenchymal stem cells
Source: PeerJ. 2016 Apr 14;4:e1907. doi: 10.7717/peerj.1907 (PMC4841220; doi:10.7717/peerj.1907)
Supplement: Data S1 [file peerj-04-1907-s001.pdf]

Percentage of positive cells following transfection with the pmaxGFP™ plasmid, as determined by flow cytometry

% GFP + CELLS	EXPERIMENT 1	EXPERIMENT 2	EXPERIMENT 3	N=3
Replicate 1	63,72	54,02	61,00	
Replicate 2	46,54	56,03	71,64	
Replicate 3	45,58	57,32	58,04	
Mean	51,95	55,79	63,56	57,10
SD	10,21	1,66	7,15	5,92

Cell Viability (%) following transfection with the the pmaxGFP™ plasmid, as determined by a modified Alamar Blue Assay

% VIABILITY	EXPERIMENT 1	EXPERIMENT 2	EXPERIMENT 3	N=3
Replicate 1	33,89	52,75	52,29	
Replicate 2	44,01	55,76	50,06	
Replicate 3	54,87	57,93	52,01	
Mean	44,26	55,48	51,45	50,40
SD	10,49	2,60	1,22	5,69

Cell Recovery (%)

% GFP + CELLS	EXPERIMENT 1	EXPERIMENT 2	EXPERIMENT 3	N=3
Replicate 1	33,33	35,83	40,00	
Replicate 2	33,33	43,33	39,17	
Replicate 3	54,17	48,33	35,00	
Mean	40,28	42,50	38,06	40,28
SD	12,03	6,29	2,68	4,71

Stability and persistence of transgene expression (%GFP positive cells)

% GFP + CELLS	EXPERIMENT 1			EXPERIMENT 2			EXPERIMENT 3		
	24 Hours	7 Days	14 Days	24 Hours	7 Days	14 Days	24 Hours	7 Days	14 Days
Replicate 1	63,72	56,86	13,00	54,02	42,91	18,24	61,00	50,73	19,44
Replicate 2	46,54	39,29	7,86	56,03	41,20	17,38	71,64	56,06	21,19
Replicate 3	45,58	35,63	8,02	57,32	43,11	21,38	58,04	42,85	16,77
Mean	51,95	43,93	9,63	55,79	42,41	19,00	63,56	49,88	19,13
SD	10,21	11,35	2,92	1,66	1,05	2,11	7,15	6,65	2,23

	N=3		
% GFP + CELLS	24 Hours	7 Days	14 Days
Mean	57,10	45,41	15,92
SD	5,92	3,95	5,45

Percentage of positive cells following transfection with the pIRES2-EGFP-FIX plasmid, as determined by flow cytometry

	DONOR 1					
% GFP + CELLS	EXPERIMENT 1	EXPERIMENT 2	EXPERIMENT 3	EXPERIMENT 4	EXPERIMENT 5	EXPERIMENT 6
Replicate 1	26,93	31,69	18,57	23,30	29,51	34,53
Replicate 2	31,85	37,91	25,87	34,63	31,24	34,90
Replicate 3	41,00					
Mean	33,26	37,91	22,22	28,97	30,38	34,72
SD	7,14	4,40	5,16	8,01	1,22	0,26

	DONOR 2		
% GFP + CELLS	EXPERIMENT 1	EXPERIMENT 2	EXPERIMENT 3
Replicate 1	44,80	37,51	38,55
Replicate 2	41,38	42,36	42,34
Replicate 3	37,91	50,60	41,32
Mean	41,36	43,49	40,74
SD	3,45	6,62	1,96

	DONOR 3			
% GFP + CELLS	EXPERIMENT 1	EXPERIMENT 2	EXPERIMENT 3	EXPERIMENT 4
Replicate 1	32,05	23,84	38,36	27,08
Replicate 2	31,48	32,89	42,81	29,33
Replicate 3	23,22		38,68	35,22
Mean	28,92	28,37	39,95	30,54
SD	4,94	6,40	2,48	4,20

% GFP + CELLS	DONOR 1	DONOR 2	DONOR 3	N=3
EXPERIMENT 1	33,26	41,36	28,92	
EXPERIMENT 2	34,80	43,49	28,37	
EXPERIMENT 3	22,22	40,74	39,95	
EXPERIMENT 4	28,97		30,54	
EXPERIMENT 5	30,38			
EXPERIMENT 6	34,72			
Mean	30,72	41,86	31,94	34,84
SD	4,37	1,44	5,42	6,11

% Cell Viability following transfection with the the pIRES2-EGFP-FIX plasmid, as determined by a modified Alamar Blue Assay

	DONOR 1					
% VIABILITY	EXPERIMENT 1	EXPERIMENT 2	EXPERIMENT 3	EXPERIMENT 4	EXPERIMENT 5	EXPERIMENT 6
Replicate 1	39,48	32,19	35,63	33,98	39,87	33,63
Replicate 2	42,49	25,39	45,73	29,51	39,58	30,21
Replicate 3	37,40	29,61				
Mean	39,79	29,06	40,68	31,75	39,73	31,92
SD	2,56	3,43	7,14	3,16	0,21	2,42

	DONOR 2		
% VIABILITY	EXPERIMENT 1	EXPERIMENT 2	EXPERIMENT 3
Replicate 1	25,35	27,55	21,48
Replicate 2	24,21	22,78	20,10
Replicate 3	21,84	15,45	17,26
Mean	23,80	21,93	19,61
SD	1,79	6,09	2,15

	DONOR 3			
% VIABILITY	EXPERIMENT 1	EXPERIMENT 2	EXPERIMENT 3	EXPERIMENT 4
Replicate 1	28,37	40,21	33,67	42,72
Replicate 2	24,28	24,39	24,40	42,46
Replicate 3	30,57		28,72	34,86
Mean	27,74	32,30	28,93	40,01
SD	3,19	11,19	4,64	4,46

% VIABILITY	DONOR 1	DONOR 2	DONOR 3	N=3
EXPERIMENT 1	39,79	23,80	27,74	
EXPERIMENT 2	29,06	21,93	32,30	
EXPERIMENT 3	40,68	19,61	28,93	
EXPERIMENT 4	31,75		40,01	
EXPERIMENT 5	39,73			
EXPERIMENT 6	31,92			
Mean	35,49	21,78	32,25	29,84
SD	4,68	2,10	5,53	7,16

% EGFP positive cells perfused in mice

	% GFP + CELLS	SD
Experiment 1	28,31	
Experiment 2	34,35	
Experiment 3	29,63	
Experiment 4	27,54	
Experiment 5	41,64	
N=5	32,29	5,86

Concentration of factor IX secreted to culture medium by hASCs transfected with pIRES2-EGFP-FIX plasmid

	DONOR 1 (CONTROL)					
	EXPERIMENT 1	EXPERIMENT 2	EXPERIMENT 3	EXPERIMENT 4	EXPERIMENT 5	EXPERIMENT 6
Replicate 1	0,001	0,079	0,000	0,040	0,004	0,004
Replicate 2	0,008	0,054	0,000	0,000	0,013	0,013
Replicate 3	0,026	0,030	0,054	0,000	0,021	0,000
Mean	0,01	0,05	0,02	0,01	0,01	0,01
SD	0,01	0,02	0,03	0,02	0,01	0,01

	DONOR 1 (NUCLEOFECTED)					
	EXPERIMENT 1	EXPERIMENT 2	EXPERIMENT 3	EXPERIMENT 4	EXPERIMENT 5	EXPERIMENT 6
Replicate 1	29,49	30,98	33,42	33,55	57,24	45,95
Replicate 2	36,40	32,57	49,31	42,68	52,03	48,91
Replicate 3	37,14	29,48				
Mean	34,34	31,01	41,37	38,12	54,64	47,43
SD	4,22	1,54	11,24	6,45	3,68	2,09

	DONOR 2 (CONTROL)		
	EXPERIMENT 1	EXPERIMENT 2	EXPERIMENT 3
Replicate 1	0,013	0,021	0,000
Replicate 2	0,054	0,000	0,084
Replicate 3	0,017	0,000	0,000
Mean	0,03	0,01	0,03
SD	0,02	0,01	0,05

	DONOR 2 (NUCLEOFECTED)		
	EXPERIMENT 1	EXPERIMENT 2	EXPERIMENT 3
Replicate 1	34,05	57,19	33,39
Replicate 2	30,00	48,02	32,27
Replicate 3	24,37	42,86	29,34
Mean	29,47	49,36	31,66
SD	4,86	7,26	2,09

	DONOR 3 (CONTROL)			
	EXPERIMENT 1	EXPERIMENT 2	EXPERIMENT 3	EXPERIMENT 4
Replicate 1	0,000	0,000	0,001	0,000
Replicate 2	0,000	0,000	0,008	0,000
Replicate 3	0,000	0,000	0,000	0,000
Mean	0,00	0,00	0,00	0,00
SD	0,00	0,00	0,00	0,00

	DONOR 3 (NUCLEOFECTED)			
	EXPERIMENT 1	EXPERIMENT 2	EXPERIMENT 3	EXPERIMENT 4
Replicate 1	40,90	50,61	61,46	73,74
Replicate 2	37,89	48,26	69,32	71,83
Replicate 3	32,06		57,86	70,90
Mean	36,95	49,43	62,88	72,16
SD	4,49	1,66	5,86	1,45

	CONTROL				NUCLEOFECTED			
	DONOR 1	DONOR 2	DONOR 3	N=3	DONOR 1	DONOR 2	DONOR 3	N=3
EXPERIMENT 1	0,01	0,03	0,00		34,34	29,47	36,95	
EXPERIMENT 2	0,05	0,01	0,00		31,01	49,36	49,43	
EXPERIMENT 3	0,02	0,03	0,00		41,37	31,66	62,88	
EXPERIMENT 4	0,01		0,00		38,12		72,16	
EXPERIMENT 5	0,01				54,64			
EXPERIMENT 6	0,01				47,43			
Mean	0,02	0,02	0,00	0,01	41,15	36,83	55,35	44,45
SD	0,02	0,01	0,00	0,01	7,96	10,90	15,41	9,69

Transfected cells obtained from one of the donors secreted a larger amount of FIX

	EXPERIMENT 1	EXPERIMENT 2	N=2
Replicate 1	59,48	71,13	
Replicate 2	79,15	79,11	
Replicate 3	73,81	68,49	
Mean	70,81	72,91	71,86
SD	10,17	5,53	1,48

Levels of hepatic marker enzymes ALT and AST, as determined 24 hours after administration of APAP

	ALT			AST		
	Replicates	Mean	SD	Replicates	Mean	SD
500 mg/kg	23	26,00	4,36	94	129,00	32,42
	31			158		
	24			135		
Fast + vehicle	38	34,00	4,00	178	110,00	59,19
	34			82		
	30			70		
Fast + 400 mg/kg	64	73,33	31,07	400	404,67	219,04
	108			626		
	48			188		
Fast + 500 mg/kg	118	132,00	19,80	472	618,00	206,48
	146			764		

Concentration of factor IX in plasma of NSG mice after injection of hASCs transfected with pIRES2-EGFP-FIX plasmid

		FIX (ng/mL)	Mean	SD
48 hours	Mice 1	2,58	2,68	0,69
	Mice 2	1,45		
	Mice 3	3,42		
	Mice 4	3,18		
	Mice 5	2,85		
	Mice 6	2,59		
5 days	Mice 1	1,76	1,31	0,76
	Mice 2	2,63		
	Mice 3	1,13		
	Mice 4	0,76		
	Mice 5	0,63		
	Mice 6	0,97		
Control	Mice 1	0,05	0,02	0,02
	Mice 2	0,00		
	Mice 3	0,01		
