# Supplementary material for: Preliminary study on non-viral transfection of F9 (factor IX) gene by nucleofection in human adipose-derived mesenchymal stem cells
Source: PeerJ. 2016 Apr 14;4:e1907. doi: 10.7717/peerj.1907 (PMC4841220; doi:10.7717/peerj.1907)
Supplement: Supplemental Information 2 [file peerj-04-1907-s002.pdf]

	Sequence (5'->3')	Length	Start	Stop	Tm	GC%	Self complementarity	Self 3' complementarity
**Forward primer**	CTGCTCCTTCCATCTGCCTG	20	8425	8444	60.46	60.00	2.00	1.00
**Reverse primer**	GGATGCTTCCTCCAACTGCT	20	8534	8515	60.03	55.00	6.00	1.00
**Product length**	110							

Products on potentially unintended templates

>NM_001033263.4 Mus musculus ArfGAP with GTPase domain, ankyrin repeat and PH domain 2 (Agap2), mRNA

```
product length = 594
Forward primer  1     CTGCTCCTTCCATCTGCCTG  20
Template        1847  .......CAG..G..C....  1828

Reverse primer  1     GGATGCTTCCTCCAACTGCT  20
Template        1254  T.GC..C.......C.....  1273
```

>NM_001083318.1 Mus musculus ets variant gene 3 (Etv3), transcript variant 1, mRNA

```
product length = 1858
Forward primer  1     CTGCTCCTTCCATCTGCCTG  20
Template        1876  ...TC..C...TG.......  1895

Reverse primer  1     GGATGCTTCCTCCAACTGCT  20
Template        3733  A..GC.......T.C.....  3714
```

>NM_001039939.1 Mus musculus additional sex combs like 1 (Asxl1), mRNA

```
product length = 1264
Forward primer  1     CTGCTCCTTCCATCTGCCTG  20
Template        4268  ...T..TC.G.........T  4249

Reverse primer  1     GGATGCTTCCTCCAACTGCT  20
Template        3005  ...CA.C.......G.A...  3024
```

>NM_001177437.1 Mus musculus maestro heat-like repeat family member 4 (Mroh4), mRNA

```
product length = 437
Forward primer  1     CTGCTCCTTCCATCTGCCTG  20
Template        1416  ...TC.AG.......A....  1435

Forward primer  1     CTGCTCCTTCCATCTGCCTG  20
Template        1852  ...........TGAA...A.  1833
```

>NM_001168390.1 Homo sapiens chromosome 10 open reading frame 105 (C10orf105), transcript variant 2, mRNA

```
product length = 3292
Forward primer  1     CTGCTCCTTCCATCTGCCTG  20
Template        1021  G....G.CAG..........  1040

Forward primer  1     CTGCTCCTTCCATCTGCCTG  20
Template        4312  .CT..G.C.........A..  4293
```

>NM_001144963.1 Homo sapiens nuclear factor of kappa light polypeptide gene enhancer in B-cells inhibitor-like 1 (NFKBIL1), transcript variant 4, mRNA

```
product length = 823
Forward primer  1     CTGCTCCTTCCATCTGCCTG  20
Template        1231  ..TAC......T.......C  1212

Reverse primer  1    GGATGCTTCCTCCAACTGCT  20
Template        409  .AT.T........CG.....  428
```

>NM_001144962.1 Homo sapiens nuclear factor of kappa light polypeptide gene enhancer in B-cells inhibitor-like 1 (NFKBIL1), transcript variant 3, mRNA

```
product length = 868
Forward primer  1     CTGCTCCTTCCATCTGCCTG  20
Template        1276  ..TAC......T.......C  1257

Reverse primer  1    GGATGCTTCCTCCAACTGCT  20
Template        409  .AT.T........CG.....  428
```

>NM_001144961.1 Homo sapiens nuclear factor of kappa light polypeptide gene enhancer in B-cells inhibitor-like 1 (NFKBIL1), transcript variant 2, mRNA

```
product length = 823
Forward primer  1     CTGCTCCTTCCATCTGCCTG  20
Template        1284  ..TAC......T.......C  1265

Reverse primer  1    GGATGCTTCCTCCAACTGCT  20
Template        462  .AT.T........CG.....  481
```

>NM_005007.3 Homo sapiens nuclear factor of kappa light polypeptide gene enhancer in B-cells inhibitor-like 1 (NFKBIL1), transcript variant 1, mRNA

```
product length = 868
Forward primer  1     CTGCTCCTTCCATCTGCCTG  20
Template        1329  ..TAC......T.......C  1310

Reverse primer  1    GGATGCTTCCTCCAACTGCT  20
Template        462  .AT.T........CG.....  481
```
